# Editorial: Psychosocial Risks and Health at Work From a Gender Perspective

**DOI:** 10.3389/fpsyg.2019.01392

**Published:** 2019-06-25

**Authors:** Eva Cifre, María Vera

**Affiliations:** ^1^Research Group Género, Salud y Trabajo, Department of Developmental and Social Psychology and Methodology, Universitat Jaume I, Castellón, Spain; ^2^Department of Education and Social Psychology, Universidad Pablo de Olavide, Seville, Spain

**Keywords:** psychosocial risk, health, work, gender, sex, gender identidy, mental health - state of emotional and social well-being

The number of working women is continuously increasing and becoming more significant (78.1% of men and 66.6% of women in Europe EU28, European Commission, 2018). This has been reflected in the presence of a gender dimension on the occupational health psychology (OHP). However, this gender dimension has not been included in OHP research or practice, which mostly still considers men as the single measure (i.e., *androcentrism*). Thus, there is a clear need to consider gender effects on OHP issues. This special issue seeks to inform the global community about advances on it.

A comprehensive international, multifactorial, interdisciplinary, and intentional effort is crucial to understand how different aspects of the work-life are influencing the occupational psychosocial health in the same way as or in a different way from different genders. This research topic in *Frontiers in Psychology* includes 11 papers from across the globe that address psychosocial health at work from a holistic point of view, considering individual, work, social, and organizational factors that influence women's and men's psychosocial health at work. In this vein, these papers cover a range of topics and address gender issues going further sex–gender dichotomy (men–women), including top issues in the area, such as the LGBT community and gender identity.

The present e-book consists of different types of papers (one conceptual analysis paper, one hypothesis and theory paper, and nine original research) that combine different methodologies to shed new insights regarding this topic, both by comparing sex and gender. And not only from psychology, but also from other related fields (i.e., management, sociology, education, and medicine).

The e-book starts with a review paper by Gartzia et al. who analyze research on gender stereotypes and identity and emotions as psychosocial antecedents of organizational stress in the PsycINFO database during the period 1980–2017. Authors conclude that androgynous individuals may have the potential to develop a wide range of emotional competencies that are required to deal with and improve emotional experiences at work. Gender identity also seems to play a crucial role to perceive employability in youngsters in Spain. Cifre et al.'s original study shows that masculine traits are still the most searched by organizations, independently of their sex (men–women), so young individuals with more masculine characteristics and less feminine ones are those that feel more employable.

Several of the contributions focus on work–family conflict (WFC), a phenomenon in which sex, gender identity, and organizational factors interact. Carvalho et al. find among a multigroup study on Portuguese employees that job demands and lack of control could contribute to employees' stress and, once individuals' energy was drained, WFC could emerge, affecting negatively mental health. No differences between men and women were found. Following with mental health, Zhou et al. found on a sample of Chinese female employees that WFC affects the level of self-reported mental health, and this relationship functioned through the two sequential mediators of negative affect and perceived stress.

In the same vein, Emanuel et al. explored, among Italian heterosexual couples, the relationship between one worker's perceived job insecurity and family life satisfaction of both the person and his/her partner. Results show a spillover effect. These effects were similar among men and women, showing that there are some changes among men and women in perceiving their family and work roles, as well as in the degree in which these roles are central to their identity. However, these differences between women and men still appear when we focus on the family domain. In this line, Cerrato and Cifre confirm, in a sample of Spanish employees, inequality in household chores, as men focus on traditionally masculine duties for men and feminine ones for women. Also, women's involvement is more than double than their partners, which has negative consequences in the family sphere for women and the workplace for men. Then, the traditional gender role model seems still active. Finally, Barnard explores the work–life experiences of South African women in their midlife through focus groups, based on the socioanalytic method of social dream drawing. Findings show how women's projective identification with outdated gender role norms may perpetuate a gendered notion of work–life balance, which consistently challenges their well-being.

Furthermore, three papers focus on organizational factors affecting occupational health at work of women and men. Firstly, Kowalczuk et al. concentrate on the phenomenon of horizontal segregation by assessing the influence of psychosocial hazards as a factor affecting the presence of men in the nursing profession. The authors found, among Polish nurses, that the profession was assessed more negatively by men than by women. The more significant psychosocial hazards experienced by men nurses may affect the low representation of men among practicing nursing staff. Secondly, García-Izquierdo et al. focus on the phenomenon of vertical segregation, examining the presence of female directors both at board meetings and an audit and remuneration committees and CEO pay and remuneration. Results corroborate that the incorporation of women appears to exert a positive effect in terms of higher wage moderation, and restraint in the use of long-term variable remuneration systems. Finally, Conesa and González-Ramos, through interviews with Spanish women and men researchers, highlight discriminatory practices toward women academics, which create psychological harm and feelings of being unwelcome, putting their career progression at risk.

Finally, García Johnson and Otto develop an integrative model of gender equality in the workplace for HRM academics and practitioners, beyond white, heterosexual, and cisgender women. Authors underscore the importance of industry–university collaboration and offer a starter's toolkit that includes suggestions for diagnosis, intervention, and applied research on gender-based discrimination and harassment.

To sum up, these 11 papers included in this e-book provide new avenues for future research to understand how different work factors affect men and women at work, with the final aim of contributing to a healthier and fairer society for all of us.

## Author Contributions

All authors listed have made a substantial, direct and intellectual contribution to the work, and approved it for publication.

### Conflict of Interest Statement

The authors declare that the research was conducted in the absence of any commercial or financial relationships that could be construed as a potential conflict of interest.
